# Short-Term Snow Removal Alters Fungal but Not Bacterial Beta Diversity and Structure during the Spring Snowmelt Period in a Meadow Steppe of China

**DOI:** 10.3390/jof8030234

**Published:** 2022-02-26

**Authors:** Hengkang Xu, Nan Liu, Yingjun Zhang

**Affiliations:** 1 College of Grassland Science and Technology, China Agricultural University, 2 Yuan Ming Yuan West Road, Haidian District, Beijing 100193, China; xuhengkang@cau.edu.cn (H.X.); liunan@cau.edu.cn (N.L.); 2 Key Laboratory of Grassland Management and Rational Utilization, Ministry of Agriculture and Rural Affairs, Beijing 100193, China

**Keywords:** climate change, snow cover, soil microfungi, soil microbial communities, community diversity, illumina sequencing

## Abstract

Global climate change is altering the amounts of ice and snow in winter, and this could be a major driver of soil microbial processes. However, it is not known how bacterial and fungal communities will respond to changes in the snow cover. We conducted a snow manipulation experiment to study the effects of snow removal on the diversity and composition of soil bacterial and fungal communities. A snow manipulation experiment was carried out on the meadow steppe in Hulunbuir, Inner Mongolia, China, during the winter period October 2019–March 2020. Soil samples were collected from the topsoil (0–10 cm) in mid-March 2020 (spring snowmelt period). Snow removal significantly reduced soil moisture and soil ammonium concentration. Lower snow cover also significantly changed the fungal community structure and beta diversity. Snow removal did not affect the bacterial community, indicating that fungal communities are more sensitive to snow exclusion than bacterial communities. The relative importance analysis (using the Lindeman–Merenda–Gold method) showed that available nitrogen (AN), soil water content (SWC), total organic carbon (TOC), microbial biomass carbon (MBC), and microbial biomass nitrogen (MBN) together explained 94.59% of the variation in soil fungal beta diversity, where AN was identified as the most important predictor. These finding provide insights into potential impacts of climate warming and associated reduced snow cover on soil microbial communities and processes.

## 1. Introduction

Climate change is predicted to reduce the thickness of the snow cover in many cold-climate regions in the world [1,2]. Reduced snow increases soil freezing depth, increases soil temperature variability, and reduces soil moisture [3,4]. Soil microclimatic conditions (e.g., temperature, moisture, frost intensity, and duration) regulate soil microbial communities in cold climate ecosystems [3,5,6,7]. Changes in soil microclimatic conditions will likely have severe ecological impacts due to the importance of winter snow for meadow ecosystem functioning. Water availability is a prior limiting factor for primary production, and snowfall in winter is an important source of water in the meadow steppe in Inner Mongolia. Hence, reduced snow cover associated with winter climate change can produce drastic changes in soil temperature and moisture during the spring snowmelt, which may in turn influence soil biological processes, especially those related to soil bacterial and fungal communities [8,9,10].

Soil microbial communities regulate global biogeochemical cycles and respond rapidly to changing environmental conditions [11]. The diversity and composition of bacterial and fungal communities play key roles in soil carbon and nutrient cycling in cold climates [12,13]. Therefore, it is important to understand how bacterial and fungal communities respond to snow cover change. The effect of snow changes on soil temperature and water content during the freeze–thaw period may directly change the bacterial and fungal communities [8,9]. The influences of snow depth on soil bacterial and fungal communities have been previously evaluated [8,9,10,14]. However, changes in bacterial and fungal community diversity and structure in response to variations in snow cover are inconsistent. For example, bacterial diversity was generally not particularly sensitive to the absence of snow cover in Tibetan spruce forest soil and subalpine grassland [8,15]. On the contrary, as snow depths increased, bacterial diversities greatly increased in the Jinchuan wetland or alpine meadows of China [16,17]. In addition, a long-term increase in snow depth led to compositional changes in arctic ectomycorrhizal (ECM) fungal communities [18], while fungal communities in acidic northern boreal forest soil may be less sensitive to the direct effects of changing snow cover [9]. The majority of these studies focused on forest or tundra; it is not known how different members of the bacterial and fungal communities respond to snow changes on the northern meadow steppe of China.

Snow cover change may also induce significant changes in soil biochemical properties (e.g., N availability), and thereby may mediate climate-induced shifts in soil microbial communities [10,19]. Snowmelt can create a longer anaerobic period during the spring thaw, which stimulates microbial denitrifying activity and results in a large pulse of N_2_O emissions and N leaching [12,20,21]. Change in N could potentially influence the relative importance of deterministic and stochastic processes, and therefore, the trajectories of soil microbial community assembly. For example, a recent study showed that N input was correlated with soil bacterial diversity but not fungal beta diversity [22]. In addition, snow cover changes may have a greater impact on fungal community composition, which is closely related to nutrient cycling and plant growth [23,24]. For example, the arbuscular mycorrhizal fungal (AMF) abundance increased with snow depth in temperate steppes [24], which could facilitate nutrient transfer from the soil to plants [23]. In addition, a long-term increase in snow depth can lead to compositional changes in arctic ECM fungal communities [18]. Therefore, it is critical to explore the influence of environmental factors (i.e., temperature and moisture) and soil biochemical variables (i.e., soil available nitrogen) on the structure and function of soil bacterial and fungal communities and to construct a model that explicitly considers microbial diversity.

In this study, by removing snow in winter and changing the soil environment during freezing and thawing, we evaluated the immediate effects of snow exclusion on the diversity and composition of soil bacterial and fungal communities during the spring snowmelt on the meadow steppe of China. We hypothesized that (1) snow exclusion will affect the fungal and bacterial communities via changes in environmental and/or soil biochemical factors and (2) short-term snow exclusion will affect bacterial community and fungal community to different degrees due to their different sensitivity to climatic variables.

## 2. Materials and Methods

### 2.1. Site Description

A snow manipulation experiment was carried out on the meadow steppe in Hulunbuir, Inner Mongolia, China (49°20′–49°26′ N, 119°55′–120°9′ E), during the winter period October 2019–March 2020. The elevation of the study area varies from 628 to 649 m a.s.l. It has a temperate continental monsoon climate with mean (1951–2018) annual precipitation of 354.5 mm, with 75.0% of precipitation falling from June through September. Mean annual temperature was −1.2 °C, ranging from −26.0 °C in January to 20.0 °C in July [25]. Snow cover usually lasts from late October to late March of the following year. The site has a dark chestnut soil (Calcic Chernozem according to ISSS Working Group RB, 1998). In the experimental field, *Leymus chinensis* (Trin.) Tzvel., *Bromus inermis* Leyss., and *Potentilla bifurca* L. represent the dominant plant species [26].

### 2.2. Experimental Design

Our field experiment comprised ambient snow (control) and snow removal (S0) treatments, which were established in October 2019 using shelters with plastic film coverings to create the snow exclusion treatment (Figure S1). There was a total of eight plots (viz. 4 plots per treatment × 2 treatments = 8 plots). The size of the plots was 3 m × 3 m. A random block design was used in the experiment. One control plot was randomly set up in the vicinity of each snow-exclusion plot. All treatment plots were separated by at least 2 m to reduce their influence on each other. Based on Sulkava and Huhta [27] and Tan and Wu [28], four plastic film coverings (0.3 m above the soil surface at the eaves; covering an area of 3 m × 3 m) were installed in October 2019 to prevent snow accumulation on the ground. The film was made of low-density polyethylene that transmits ~80% of photosynthetically active radiation. Snow removal manipulation began on 20 October 2019, ending in mid-March 2020.

### 2.3. Microclimate

Soil moisture under different winter snow cover was measured at 10 cm soil depth during the spring snowmelt (20 March 2020). Soil samples (5 cm in diameter and 10 cm in depth) were dried at 105 °C for 48 h to a constant weight to determine soil moisture content. The air temperature (1 m height) and soil temperature (10 cm depth) were measured using Thermochron DS1923 iButtons (Wdsen Electronic Technology Co., Ltd., Shanghai, China) at 1 h intervals every 1 h from 1 October in 2019 to 1 May in 2020. Accumulated monthly snowfall was measured during October 2019–March 2020 after each snowfall event (Figure S2a). Ambient snow depth in the control plots was measured on 15 December 2019 and 15 March 2020 with a ruler. The average of six random measurements within each plot was recorded to determine the depth of ambient snow (CK). 

### 2.4. Soil Sampling and Soil Biochemical Analyses

Soil samples were collected from the topsoil (0–10 cm) in mid-March 2020 (spring snowmelt period). Three soil cores (5 cm in diameter, 0–10 cm deep) were randomly taken at each plot and were mixed into one composite sample per plot using a soil auger. The composite samples were passed through a 2 mm sieve, and any visible living plant material was removed from the sieved soil. Subsamples of the sieved soils were stored at −80 °C and 4 °C for molecular and biochemical analyses, respectively. The total organic carbon (TOC) was measured with a TOC analyzer (Rapid CS Cube, Elementar, Norderstedt, Germany). The total carbon (TC) and total nitrogen (TN) were measured with a C/N analyzer (Rapid CS Cube, Elementar, Germany). Inorganic nitrogen (ammonium-N and nitrate-N) was extracted with 0.5 mol L^−1^ of K_2_SO_4_ and measured with a continuous flow injection analyzer (AA3 HR, SEAL Analytical GmbH, Norderstedt, Germany). Microbial biomass carbon (MBC) and microbial biomass nitrogen (MBN) were determined by comparing paired chloroform fumigation and control samples extracted in 0.5 M K_2_SO_4_ and analyzed on a Shimadzu TOC-TN device. The coefficients giving the extractable part of MBC and MBN were set at 0.45 and 0.54, respectively [29]. Available phosphorus (AP) was determined using the Olsen method, which involved adding 50 mL of Olsen’s reagent to 2.5 g of air-dried soil (soil/solution ratio of 1:20) and subsequent shaking for 30 min, then using the filtrate to determine it colorimetrically [30].

### 2.5. DNA Extraction, PCR Amplification, and Illumina MiSeq Sequencing

Microbial community genomic DNA was extracted from 8 samples (0.25 g soil) using the E.Z.N.A.^®^ soil DNA Kit (Omega Bio-tek, Norcross, GA, USA) according to manufacturer’s instructions. The DNA extract was checked on 1% agarose gel, and DNA concentration and purity were determined with NanoDrop 2000 UV-vis spectrophotometer (Thermo Scientific, Wilmington, NC, USA). The bacterial 16S rRNA gene was amplified with the primers 338F_806R, and the fugal ITS region was amplified with the primers ITS1F_ITS2 by an ABI GeneAmp^®^ 9700 PCR thermocycler (GeneAmp, 9700, ABI, USA). To profile soil bacterial communities, we amplified the V3–V4 hypervariable region of 16S rRNA gene with the primer sets 338F (5′-ACTCCTACGGGAGGCAGCAG-3′) and 806R (5′-GGACTACHVGGGTWTCTAAT-3′) [31]. For fungal communities, we amplified the ITS region with the primers sets ITS1-F (5′-CTTGGTCATTTAGAGGAAGTAA-3′) and ITS2 (5′-TGCGTTCTTCATCGATGC-3′) [32]. The PCR amplification of 16S rRNA and ITS genes were performed as follows: initial denaturation at 95 °C for 3 min, followed by 27 cycles of denaturing at 95 °C for 30 s, annealing at 55 °C for 30 s and extension at 72 °C for 45 s, and single extension at 72 °C for 10 min, and ending at 4 °C. The PCR mixtures contained 5 × TransStart FastPfu buffer 4 μL, 2.5 mM dNTPs 2 μL, forward primer (5 μM) 0.8 μL, reverse primer (5 μM) 0.8 μL, TransStart FastPfu DNA Polymerase 0.4 μL, template DNA 10 ng, and finally ddH_2_O up to 20 μL. PCR reactions were performed in triplicate. The PCR product was extracted from 2% agarose gel and purified using the AxyPrep DNA Gel Extraction Kit (Axygen Biosciences, Union City, CA, USA) according to manufacturer’s instructions and quantified using Quantus™ Fluorometer (Promega, Madison, WI, USA). Purified amplicons were pooled in equimolar and paired-end sequenced on an Illumina MiSeq PE300 platform (Illumina, San Diego, CA, USA) according to the standard protocols by Majorbio Bio-Pharm Technology Co. Ltd. (Shanghai, China). The raw 16S rRNA and ITS reads were deposited in the NCBI Sequence Read Archive (SRA) database with accession number PRJNA736681.

### 2.6. Processing of Sequencing Data

The raw 16S rRNA genes and ITS region sequencing reads were demultiplexed, quality-filtered by fastp version 0.20.0 (https://github.com/OpenGene/fastp, accessed on 15 February 2022) [33], and merged by FLASH version 1.2.7 (https://ccb.jhu.edu/software/FLASH/index.shtml, accessed on 15 February 2022) [34] with the following criteria: (i) the 300 bp reads were truncated at any site receiving an average quality score of <20 over a 50 bp sliding window, and the truncated reads shorter than 50 bp were discarded, reads containing ambiguous characters were also discarded; (ii) only overlapping sequences longer than 10 bp were assembled according to their overlapped sequence. The maximum mismatch ratio of overlap region was 0.2. Reads that could not be assembled were discarded; (iii) samples were distinguished according to the barcode and primers, and the sequence direction was adjusted, exact barcode matching, 2 nucleotide mismatches in primer matching. 

Sequenced sample libraries were processed following the DADA2 bioinformatics pipeline reported by Callahan et al. [35] using version 1.3.3 of the DADA2 R-package (R Development CoreTeam, 2015). DADA2 allows the inference of exact amplicon sequence variants (ASVs), providing several benefits over traditional OUT clustering methods [36]. Taxonomy based on 16S and ITS sequences was assigned using the SILVA v.132 (https://www.arb-silva.de/, accessed on 15 February 2022) and UNITE v.8.0 databases (https://unite.ut.ee/, accessed on 15 February 2022), respectively. Adequacy of sequencing depth after reads processing was corroborated with rarefaction curves at the ASV level.

### 2.7. Statistical Analysis

We used microbial ASVs as metrics and calculated microbial α-diversity. One-way ANOVA was used to determine the significance of the effects of snow exclusion on the following response soil variables: α-diversity of bacterial and fungal communities and MBC and MBN. Student’s *t*-test was used to determine the significance of phyla and classes of bacterial and fungal communities. Before conducting ANOVA, the normality and homoscedasticity of the residues were verified by the Kolmogorov–Smirnov test and Levene’s test, respectively. Significant differences were determined at the 0.05, 0.01, and 0.001 levels. All data are presented as mean values ± standard error (SE). Effects of snow exclusion on bacterial and fungal community structures were further tested by non-metric multidimensional scaling (NMDS) using ASVs-based Bray–Curtis. The data were analyzed on the online platform of Majorbio Cloud Platform (www.majorbio.com, accessed on 15 February 2022). Spearman correlation analysis was used to assess the relationships between the relative abundance of bacterial and fungal taxa and soil properties (i.e., soil physicochemical properties and soil moisture). FunGuild (http://www.funguild.org/, accessed on 15 February 2022) was used to annotate fungi with functional guilds. Both NMDS and Spearman analyses were performed using the VEGAN package [37] in R 3.5.2 (R Development CoreTeam, 2015). We calculated the abundance-based Bray–Curtis as the metrics of beta diversity, to quantify community compositional difference between replicate plots of the same treatment. We assessed the relative importance of soil physicochemical conditions for beta diversity of soil microbial communities, using the Lindeman–Merenda–Gold method. Relative importance values were calculated with the packages ‘relaimpo’. Other statistical analyses were performed using SPSS 20.0 (IBM Corporation, Armonk, NY, USA).

## 3. Results

### 3.1. Microclimates

The snow depth was 13.22 ± 0.15 and 30.89 ± 2.56 cm on 15 December 2019 and 15 March 2020, respectively. Compared with the control, lower snow cover reduced the average and minimal soil temperature during snow cover (Table 1 and Figure S2b). Lower snow cover on average reduced soil temperatures at the depths of 10 cm by 0.16 °C (Table 1). The minimum soil temperatures at the soil depth of 10 cm were −25.5 °C in the snow exclusion plots but were only −22 °C in the control plots (Table 1). In addition, daily variations in soil temperatures remained relatively stable in the control plots but fluctuated strongly in the snow exclusion plots (Figure S2b). Lower snow cover significantly reduced soil moisture during the spring snowmelt (20 March 2020) (Table 1). 

### 3.2. Soil C and N Pools

Snow exclusion significantly reduced the MBC and MBN and soil ammonium-N concentration during the snowmelt period (Table 1). Nitrate nitrogen (NO-N), available nitrogen (AN), total carbon (TC), total nitrogen (TN), organic carbon (SOC), C/N ratio, and available phosphorus (AP) were not affected by snow removal (Table 1).

### 3.3. Bacterial Community Structure and Species Diversity

**Figure 1:**
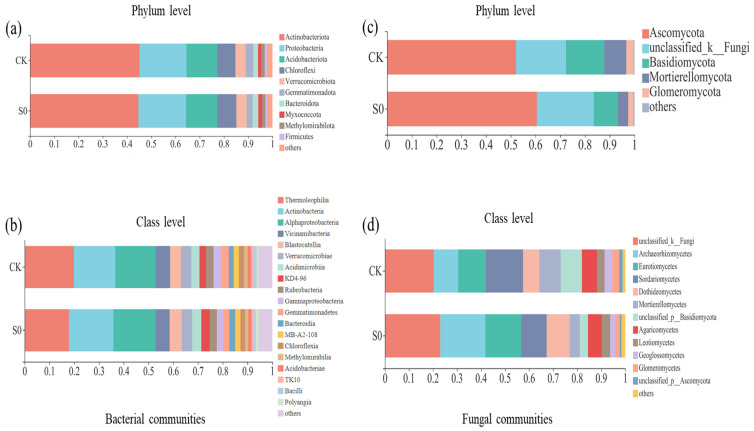
Relative abundance of bacterial communities at the phylum (**a**) and class (**b**) level, and fungal communities at the phylum (**c**) and class (**d**) level in the snow removal (S0) and control (CK) plots during the spring thawing period (March 2020). Taxa accounting for <1% were integrated into the group ‘other’.

In the bacterial community analysis, across all soil samples, a total of 24,966 high-quality sequences were identified. Each library had 199,730 reads, and 4365 ASVs were obtained. All rarefaction curves tended to approach the saturation plateau, indicating that the data volumes of the sequenced reads were reasonable (Figure S3). Sequences that could not be classified into any known group were assigned as unclassified, and groups with an average relative abundance of less than 1% were classified as ‘others’ (Figure 1). At the phylum level, *Actinobacteria* (44.7–45.1%), *Proteobacteria* (19.4–19.6%), *Acidobacteria* (12.7–12.9%), and *Chloroflexi* (7.5–7.9%) were the most dominant bacterial phyla. The relative abundances of the *Verrucomicrobia* (4.2–4.3%), *Gemmatimonadetes* (2.5–3.2%), Bacteroidetes (2.0–2.4%), *Myxococcota* (1.2–1.6%), Methylomirabilota (1.4–1.5%), and Firmicutes (0.64–1.07%) were relatively low (Figure 1a). Snow removal had little effect on bacterial community composition (Figure S4a).

**Figure 2:**
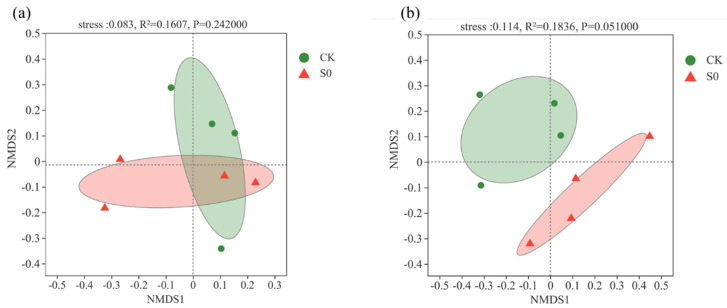
Non-metric multidimensional scaling (NMDS) of bacterial community composition (**a**) and fungal community composition (**b**) at the ASV level in control (CK) and snow exclusion (S0) plots the spring snow melting period.

At the class level, 19 bacterial classes (>1%) were observed (Figure 1b). Consistent with the phylum level, no significant differences were detected between control and snow exclusion plots at the class level (Figure S4b). Lower snow cover had no significant effect on soil bacterial community structure and alpha diversity during the spring snow melting period (Table 1; Figure 2a). 

### 3.4. Fungal Community Structure and Species Diversity

In fungal community analysis across all soil samples, a total of 60,550 high-quality sequences were identified. Each library had 38,864 reads, and a total of 2728 ASVs were obtained. Sequences were assigned as unclassified if no known group was classified, and groups with an average relative abundance less than 1% were classified as ‘other’ (Figure 1c). At the phylum level, across all sites, fungal communities were consistently dominated by *Ascomycota* (52.0–60.5%), *unclassified_k_Fungi* (20.3–23.0%), and *Basidiomycota* (9.7–15.5%). The relative abundances of *Mortierellomycota* (4.2–9.0%) and *Glomeromycota* (2.0–2.9%) were very low in all samples (Figure 1c). The relative abundance of *Mortierellomycota* was lower in the snow exclusion plots than in the control (Figure S5a; *p* < 0.05). 

At the class level, 12 fungal classes (>1%) were observed (Figure 1d). The relative abundance of *Mortierellomycetes* was higher in the control than snow exclusion plots (Figure S5b; *p* < 0.05). Lower snow cover had little effect on soil fungal α-diversity (Table 1). The fungal composition was further analyzed with NMDS at the ASVs level. The results of NMDS showed that soil fungal communities were different between control and snow exclusion plots (Figure 2b; stress = 0.114, *p* = 0.051).

### 3.5. Relationships between Bacterial and Fungal Communities and Biochemical or Environmental Factors

**Figure 3:**
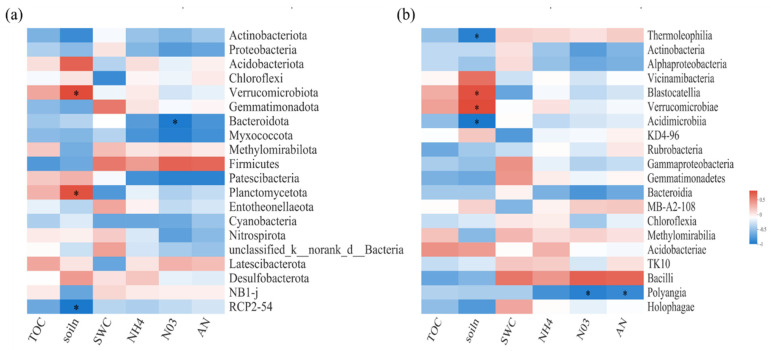
Spearman correlation heatmaps of environment factors, biochemical properties, and bacterial gene read numbers at the phylum (**a**) and class (**b**) levels. TOC: organic carbon; NH4: ammonium nitrogen; NO3: nitrate nitrogen; AN: available nitrogen; soiln: soil total nitrogen. SWC: soil water content. The color intensity in each panel indicates the relative correlation between soil properties and read numbers of each group. Statistically significant correlations are indicated with * *p* ≤ 0.05.

**Figure 4:**
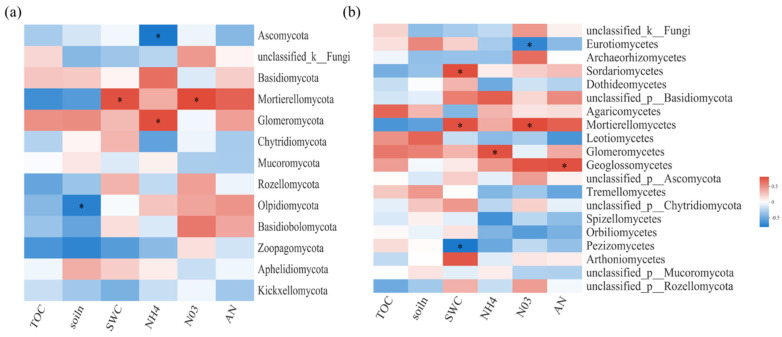
Spearman correlation heatmaps of environment factors, biochemical properties, and fungal gene read numbers at the phylum (**a**) and class (**b**) levels. TOC: organic carbon; NH4: ammonium nitrogen; NO3: nitrate nitrogen; AN: available nitrogen; soiln: total nitrogen. SWC: soil water content. The color intensity in each panel indicates the relative correlation between soil properties and read numbers of each group. Statistically significant correlations are indicated with * *p* ≤ 0.05.

Spearman correlation heatmap analysis was performed to explore the relationships between bacterial and fungal communities and biochemical or environmental factors (Figure 3 and Figure 4). For soil bacterial communities, at the phylum level, TN and NO_3_^−^N were important influence factors (Figure 3a). At the class level, SWC was significantly positively correlated with *S0134_terrestrial_group*. However, TN was significantly positively correlated with *Blastocatellia, Verrucomicrobiae, Anaerolineae,* and *Planctomycetes*, but negatively correlated with *Thermoleophilia* and *Dehalococcoidia* (Figure 3b).

**Figure 5:**
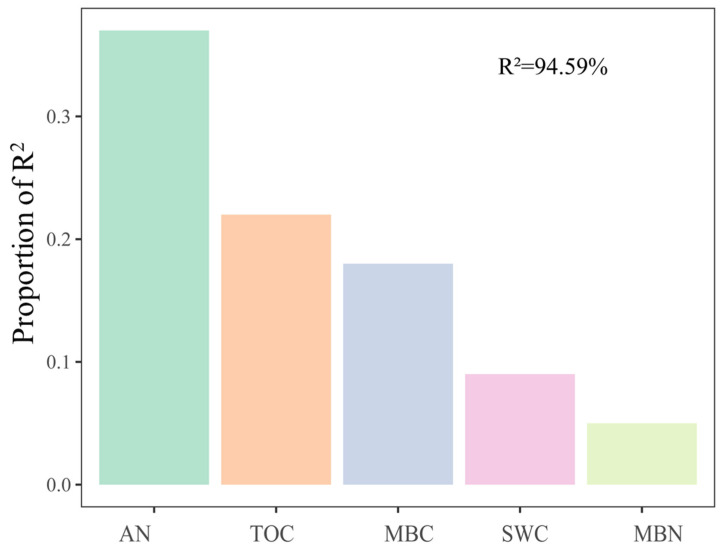
Relative importance of different predictors of fungal β-diversity. The values were calculated based on Bray–Curtis dissimilarity. Soil moisture content (SWC), available nitrogen (AN), organic carbon (TOC), microbial biomass carbon (MBC), and microbial biomass nitrogen (MBN) were used as predictors of fungi β-diversity. R^2^ indicates the total explanation. The importance values of all predictor variables were normalized to sum to 100%.

For soil fungal communities, at the class level, SWC and N were important influence factors (Figure 4a). Moreover, NO_3_^−^-N was significantly negatively correlated with *Eurotiomycetes* and significantly positively correlated with *Mortierellomycetes* and *Paraglomeromycetes* (Figure 4b). The relative importance analysis showed that AN, SWC, TOC, MBC, and MBN together explained 94.59% of the variation in soil fungal β-diversity, where AN was identified as the most important predictor (Figure 5).

## 4. Discussion

### 4.1. Impact of Snow Removal on Soil Environment

Our results show that lower snow cover decreased soil moisture content and soil temperature, consistent with our first hypothesis. During the winter (1 October to 1 May), the minimal and averaged soil temperatures were higher under the control than under the snow removal (Table 1), indicating an insulating effect of snow cover under the snow removal. This may be due to snow cover being a poor conductor of heat, with low thermal conductivity and a high thermal capacity, and it influences soil temperature by changing the energy balance, atmospheric circulation, and evaporation from the soil [38,39]. Thus, soil temperature and moisture are closely related to the depth and duration of snow cover [28,40]. For example, a 30–40 cm depth of snow cover could decouple soil temperature from air temperature, preventing the physical changes associated with soil freezing and thawing [41]. 

In addition, our findings show that lower snow cover decreased soil NH_4_^+^ concentration and meanwhile significantly decreased soil MBC and MBN during the spring snowmelt (Table 1), which is consistent with the observations found in alpine forest ecosystems [28,42] and in high alpine grassland [11]. This shows that decreases in snow cover may potentially affect soil nutrient availability and microbial properties by altering the environmental factors (i.e., soil temperature and freeze–thaw cycle) in cold ecosystems [43,44]. Changes in the microbial community further activated microbial N cycling [45]. 

There are several underlying possible mechanisms for our observations. Firstly, high substrate availability, high potential enzyme activities, and high microbial biomass carbon and nitrogen typically were found under the snow in winter [11,15,46], suggesting that high microbial biomass carbon and nitrogen during winter was associated with high potential soil enzyme activities. Thus, this would also explain the high concentrations of microbial biomass carbon and nitrogen, along with available NH_4_^+^, in snow covered soil. Secondly, previous studies have observed that higher relative abundances of genes involved in nitrification, particularly *amoA*, *amoB*, and *amoC* of AOA, and a legacy of high rates of nitrification following snowmelt, was fueled by large amounts of NH_4_^+^ accumulated during winter [11,47]. Lastly, snow cover is related to the increased intensity of soil frost and freeze–thaw cycles [48], which could enhance the release of inorganic N and the losses of N via leaching and N_2_O emissions [49]. Thus, our results show that lower snow cover significantly reduced soil ammonium-N concentration during spring snowmelt in a meadow steppe of China. However, the increases in N availability in the snow-free plots were inconsistent with other studies conducted in temperate or boreal forests [19,27,44,50]. The more frequent freeze–thaw cycles in the snow-removal treatment could accelerate the fragmentation of litter [51,52], soil aggregates [41,53,54], inactive fine roots [55,56], and soil microorganisms [57,58] and could release soluble substances into the soil, thereby enhancing the content of soil N.

### 4.2. Effect of Snow Removal on the Diversity and Structure of the Soil Bacterial Community

Lower snow cover had weak effects on bacterial community structure and diversity during spring snowmelt (Table 1). This is inconsistent with previous findings in an alpine meadow, moist acidic tundra, and temperate wetlands [10,16,17]. However, our results are consistent with the observations from Tibetan alpine forest ecosystems [8] and from temperate and boreal coniferous forest ecosystems [9], which indicated that snow cover change did not affect soil bacterial diversity and community composition. There are several underlying possible mechanisms for our observations. Most of the dominant bacterial taxa in frozen soils may have strong resistance and adaptive capabilities, thereby maintaining the stability of community structure via diverse ecological strategies [10]. For example, some specific traits (e.g., mycelium structures and sporing formations) of Actinobacteria, the dominant community (44.7–45.1%) that is insensitive to the change in snow cover, may help them to resist the extreme cold and low-nutrient conditions [59,60]. This is consistent with our observations (Figure S4b). On the other hand, high substrate affinities and the production of extracellular enzymes enhance the development of defense structure in *Alphaproteobacteria* to resist extreme conditions [61]. The above process can explain why we did not find significant effects of lower snow cover on soil bacterial communities. 

### 4.3. Effect of Snow Removal on the Diversity and Structure of the Soil Fungal Community

Lower snow cover significantly changed the fungal community structure but did not affect the bacterial and fungal community species alpha diversity. This indicated that fungal communities are more sensitive to snow exclusion than bacterial communities. Previous studies demonstrated the significant impact of snow cover changes on the soil fungal community composition and diversity in cold ecosystems [18,23,62]. For example, a long-term snow addition experiment decreased saprotrophic fungi but increased ECM fungi richness [62]. In addition, the effect of snow on the ECM fungal community was greater in dry tundra than in the moist tundra community [18]. In contrast, the lack of snow cover resulted in only slight effects on soil fungal community structure and activity in a boreal coniferous forest [9] and a Tibetan spruce forest [14] during spring snowmelt. Therefore, degree of change in fungal communities is different under different soil water conditions.

The following may account for these differences. Winter conditions (e.g., snow density, snow depth, and temperature) in different regions are different, which may cause the different responses of fungal communities. For example, a short-term mild soil freezing is not powerful enough to alter the diversity and composition of fungal communities in alpine forest soils [14]. The magnitude of soil freezing caused by snow exclusion in our study may differ from that in low-latitude alpine ecosystems and high-altitude forest ecosystems [15,19]. In addition, the soil moisture of grasslands may be lower than that of boreal coniferous forest and Tibetan spruce forest, and the fungal community is more sensitive to snow under low moisture conditions [18,24]. For example, AMF abundance against snow depth declined along with the increase in mean annual precipitation (MAP) in temperate steppes [24]. Additionally, fungal community diversity and composition varied significantly across seasons, but snow exclusion did not affect soil fungal communities in the middle of the growing season [14]. Thus, snow cover may have a greater effect on the fungal community during the freeze–thaw period, probably because the early thawing period can cause considerable leaching of nutrients. This may further shift the microbial composition due to nutrient competition [63]. As our data show, the relative importance analysis showed that AN, SWC, TOC, MBC, and MBN together explained 94.59% of the variation in soil fungal beta diversity, where soil N availability was identified as the most important predictor (Figure 5).

Additionally, fungi in different phyla and classes can respond differently to snow cover, which changes the fungi community structure and diversity. Ascomycota and Basidiomycota were the two dominant phyla in the study site. Similar findings were observed in other cold ecosystems, such as alpine meadows, desert grassland, alpine meadows, and boreal forests [14,64,65,66]. Lower snow cover significantly decreased the abundance of *Mortierellomycota* (Figure S5), indicating that these fungi have different moisture regime preferences. In addition, previous studies have shown that the relative abundance of *Ascomycota* was higher in diseased soils, while *Mortierellomycota* was higher in healthy soils [67]. We also found that saprophytes and pathogens were more responsive to snow change (Figure S6), which indicates that future changes in snow reduction may increase the risk of grassland plant and soil diseases. For the class members of *Mortierellomycota*, the relative abundance of *Mortierellomycetes* was higher in the control than snow exclusion plots, which is probably because NH_4_^+^-N was significantly positively correlated with *Mortierellomycetes*, which is consistent with the observations in subtropical forest soil [68]. 

## 5. Conclusions

This study was conducted on the meadow steppe of China and examined whether lower snow cover in winter affects soil biota during the spring snowmelt. During spring snowmelt, fungal communities were more sensitive than bacterial communities to winter snow cover changes in meadow steppe soils. The shift in fungal communities may be partly explained by environmental factors (e.g., temperature and moisture) and biochemical variables (e.g., soil N availability). Such findings may have important implications for soil microbial processes in the meadow steppe experiencing significant climate change in snowfall. Our findings suggest that a snowfall decrease associated with winter climate change might profoundly alter the phenology of soil fungal community in the meadow steppe of China. Future experiments should focus on the relationship between the plant community composition and the soil microbial community composition (e.g., bacteria, fungi) under changing snowpack.

## Figures and Tables

**Table 1:** Environmental variables (mean ± SE) of the upper soil layer (0–10 cm) in the snow removal (S0) and control (CK) plots during the spring snowmelt period (March 2020).

Treatment			
	S0	CK	F
Mean Temperature (℃)	−9.56	−9.40	-
Minimum Temperature (℃)	−25.50	−22.00	-
SWC (%)	22.8 ± 0.05	46.5 ± 0.07	3.55 ***
AN (mg/kg)	45.64 ± 4.94	61.65 ± 5.60	0.26
NH-N (mg/kg)	5.71 ± 1.42	10.26 ± 2.82	0.32 *
NO-N (mg/kg)	39.93 ± 4.89	51.39 ± 6.11	5.49
TC (g/kg)	42.71 ± 3.29	34.40 ± 1.91	1.81
TN (g/kg)	4.0 ± 0.32	3.76 ± 0.21	1.26
TOC (mg/kg)	37.22 ± 2.80	35.74 ± 1.10	0.67
AP (mg/kg)	16.63 ± 3.43	14.07 ± 1.96	0.42
MBC (mg/kg)	279.93 ± 13.97	382.64 ± 31.98	23.39 ***
MBN (mg/kg)	7.19 ± 0.51	10.89 ± 1.42	5.99 *
MBC/MBN	39.39 ± 2.87	36.78 ± 4.56	0.24
Bacterial Diversity	6.52 ± 0.10	6.43 ± 0.17	0.88
Fungal Diversity	5.0 ± 0.20	4.83 ± 0.19	0.35

Note: SWC: soil water content; AN: inorganic nitrogen; NH-N ammonium nitrogen; NO-N: nitrate nitrogen; TC: total carbon; TN: total nitrogen; TOC: organic carbon; AP: available phosphorus; MBC: microbial biomass carbon; MBN: microbial biomass nitrogen. MBC/MBN: microbial biomass carbon/nitrogen ratio. Bacterial Diversity: Bacterial Shannon Diversity Index. Fungal Diversity: Fungal Shannon Diversity Index. Significant differences between treatments are indicated with * for *p* < 0.05, *** for *p* < 0.001.

## Data Availability

Not applicable.

## Supplementary Materials

The following supporting information can be downloaded at: https://www.mdpi.com/article/10.3390/jof8030234/s1, Figure S1: Map showing the location of study site. Pictures show sampling plots before the snowfall (a) and during winter (b); Figure S2: Meteorological conditions at the study site: (a) snowfall during winter season, and (b) changes of soil temperatures (at 10 cm depth) in the control (red line) and snow removal plots (blue line); Figure S3: Rarefaction curves of bacterial (a) and fungal communities (b) tended to approach the saturation plateau, indicating that the data volume of sequenced reads was reasonable; Figure S4: Student’s *t*-test bar plot for bacterial phyla (a) and classes (b) in control and snow exclusion plots during spring snow thaw period; Figure S5: Student’s *t*-test bar plot for fungal phyla (a) and classes (b) in control and snow exclusion plots during spring snow thaw period. Statistically significant differences are indicated with: * *p* ≤ 0.05; Figure S6: Variations in composition of fungal functional groups inferred by FUNGuild. FunGuild was used to annotate fungi with functional Guild; Saprophytes and pathogens were more responsive to snow change.
