# Supplementary material for: Short-Term Snow Removal Alters Fungal but Not Bacterial Beta Diversity and Structure during the Spring Snowmelt Period in a Meadow Steppe of China
Source: J Fungi (Basel). 2022 Feb 26;8(3):234. doi: 10.3390/jof8030234 (PMC8952243; doi:10.3390/jof8030234)
Supplement: Supplementary file 1 [file jof-08-00234-s001.zip › jof-1619086-supplementary.pdf]

Short-term snow removal alters fungal but not bacterial beta diversity and structure during the spring snowmelt period in a meadow steppe of China

Hengkang Xu[a], Nan Liu[ab], Yingjun Zhang[ab]*

[a]College of Grassland Science and Technology, China Agricultural University, 2 Yuan Ming Yuan West Road, Haidian District, Beijing 100193, China.

[b]Key Laboratory of Grassland Management and Rational Utilization, Ministry of Agriculture and Rural Affairs, Beijing 100193, China

E-mail: zhangyj@cau.edu.cn (Yingjun Zhang)

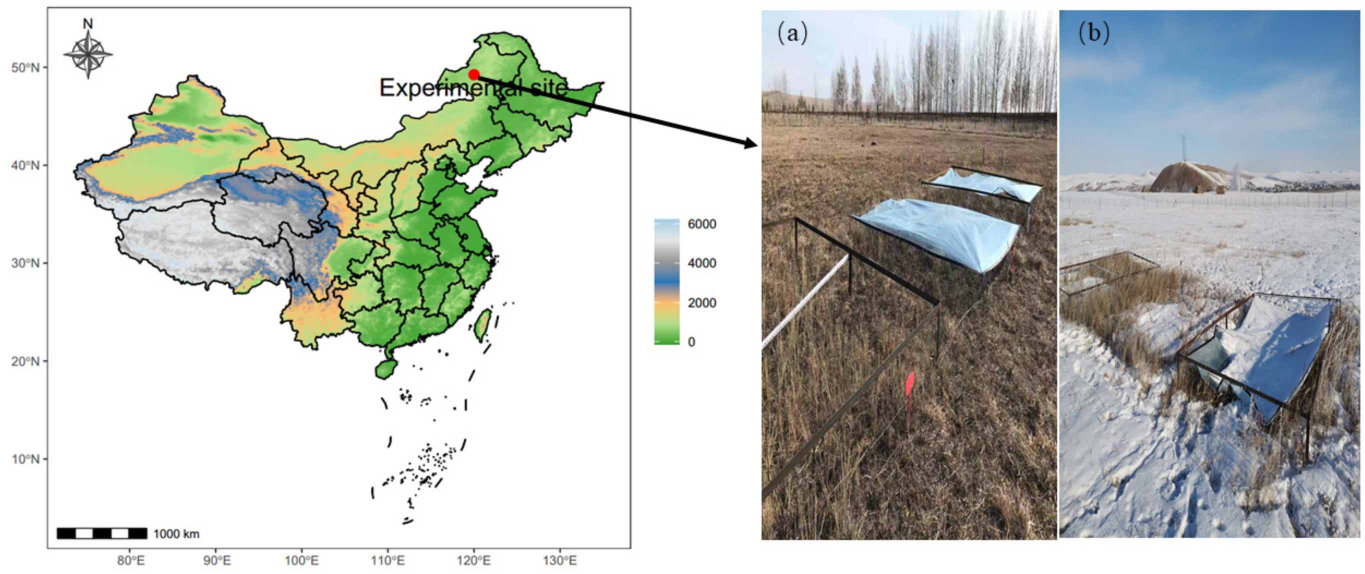

Figure S1. Map showing the location of study site. Pictures show sampling plots before the snowfall (**a**) and during winter (**b**).

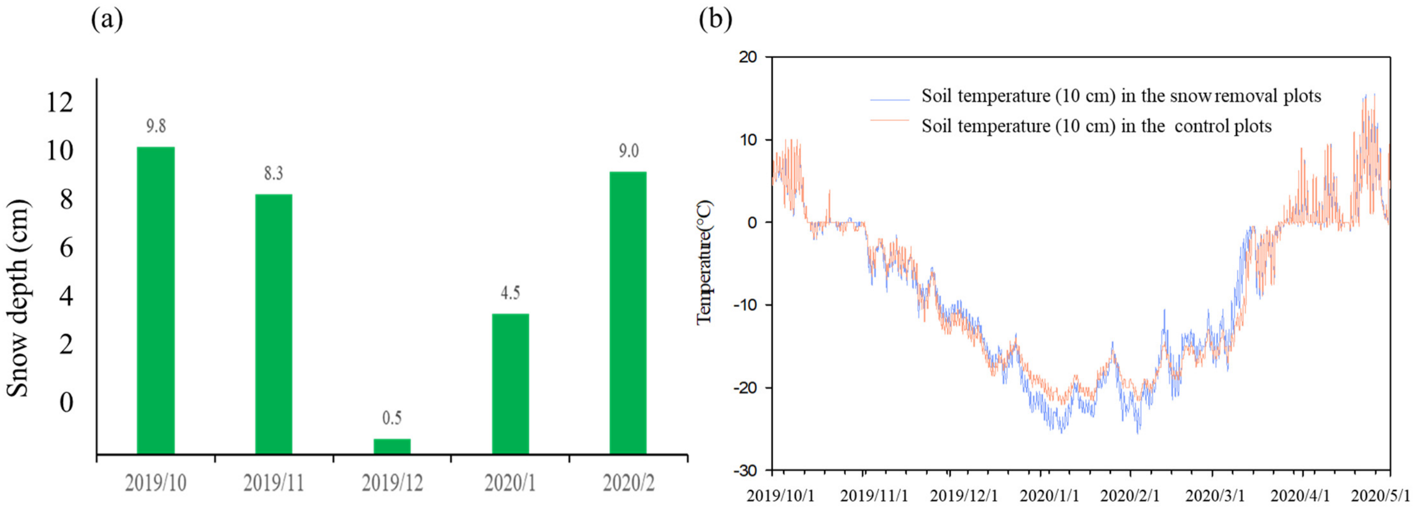

Figure S2. Meteorological conditions at the study site: a) snowfall during winter season, and b) changes of soil temperatures (at 10 cm depth) in the control (red line) and snow removal plots (blue line).

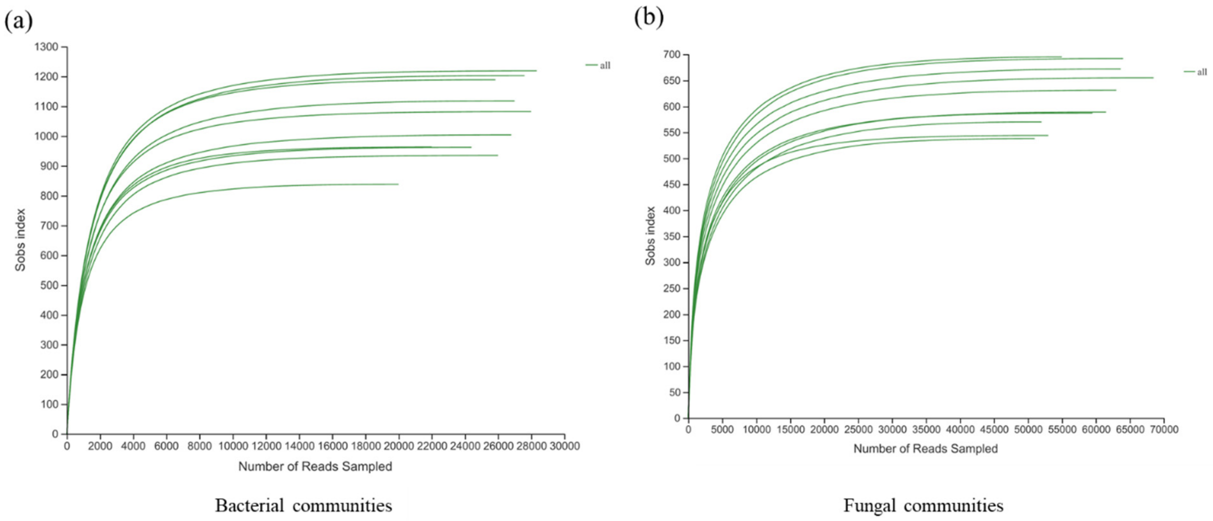

Figure S3. Rarefaction curves of bacterial **(a)** and fungal communities **(b)** tended to approach the saturation plateau, indicating that the data volume of sequenced reads was reasonable.

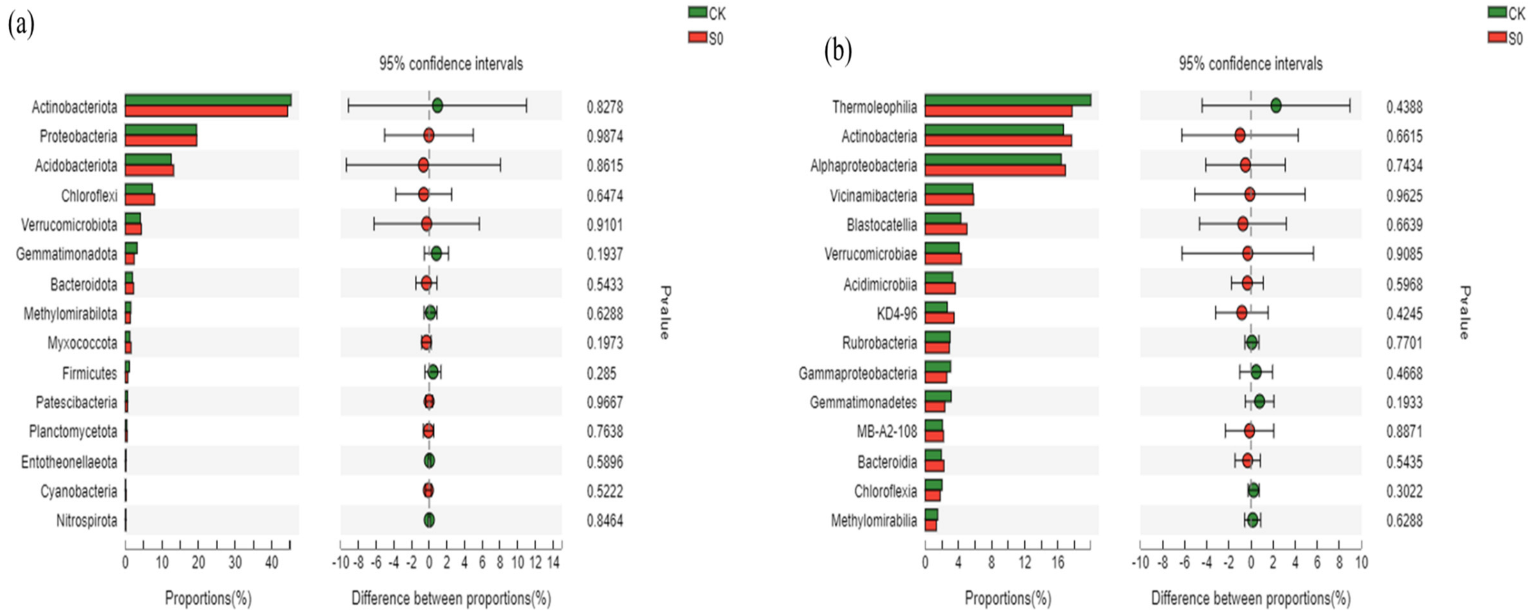

Figure S4. Student's t-test bar plot for bacterial phyla **(a)** and classes **(b)** in control and snow exclusion plots during spring snow thaw period.

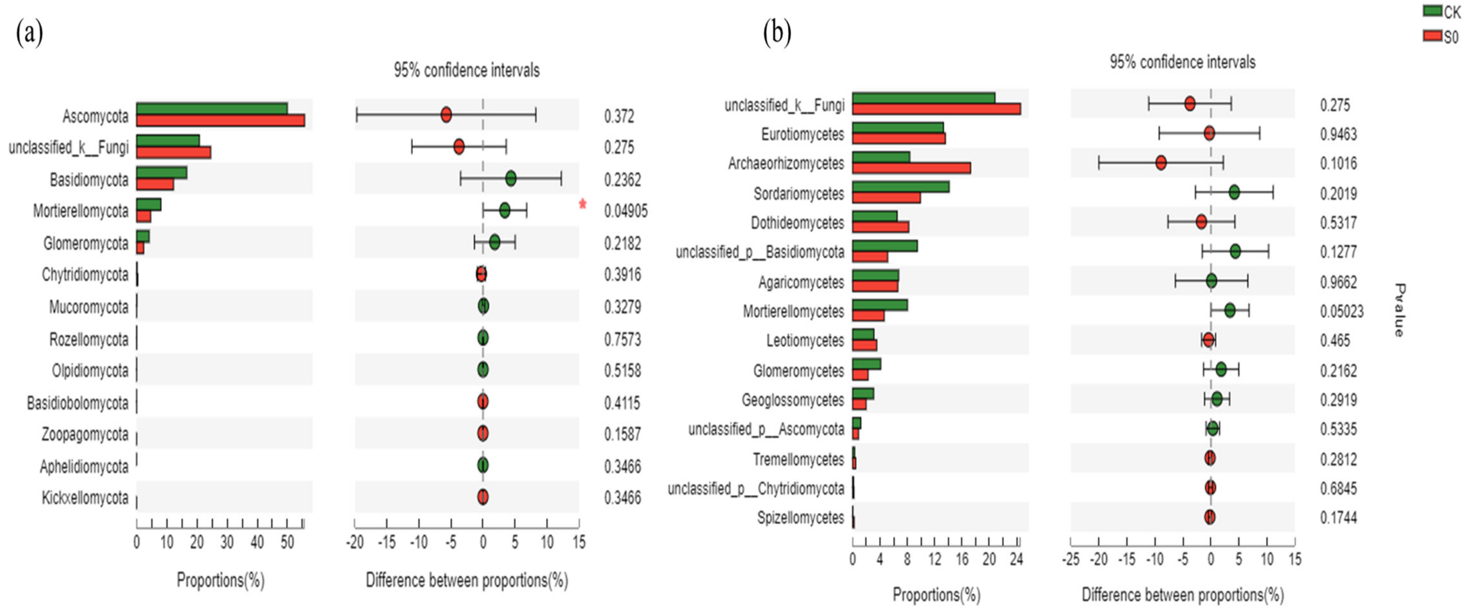

Figure S5. Student's t-test bar plot for fungal phyla **(a)** and classes **(b)** in control and snow exclusion plots during spring snow thaw period. Statistically significant differences are indicated with: * $P \leq 0.05$.

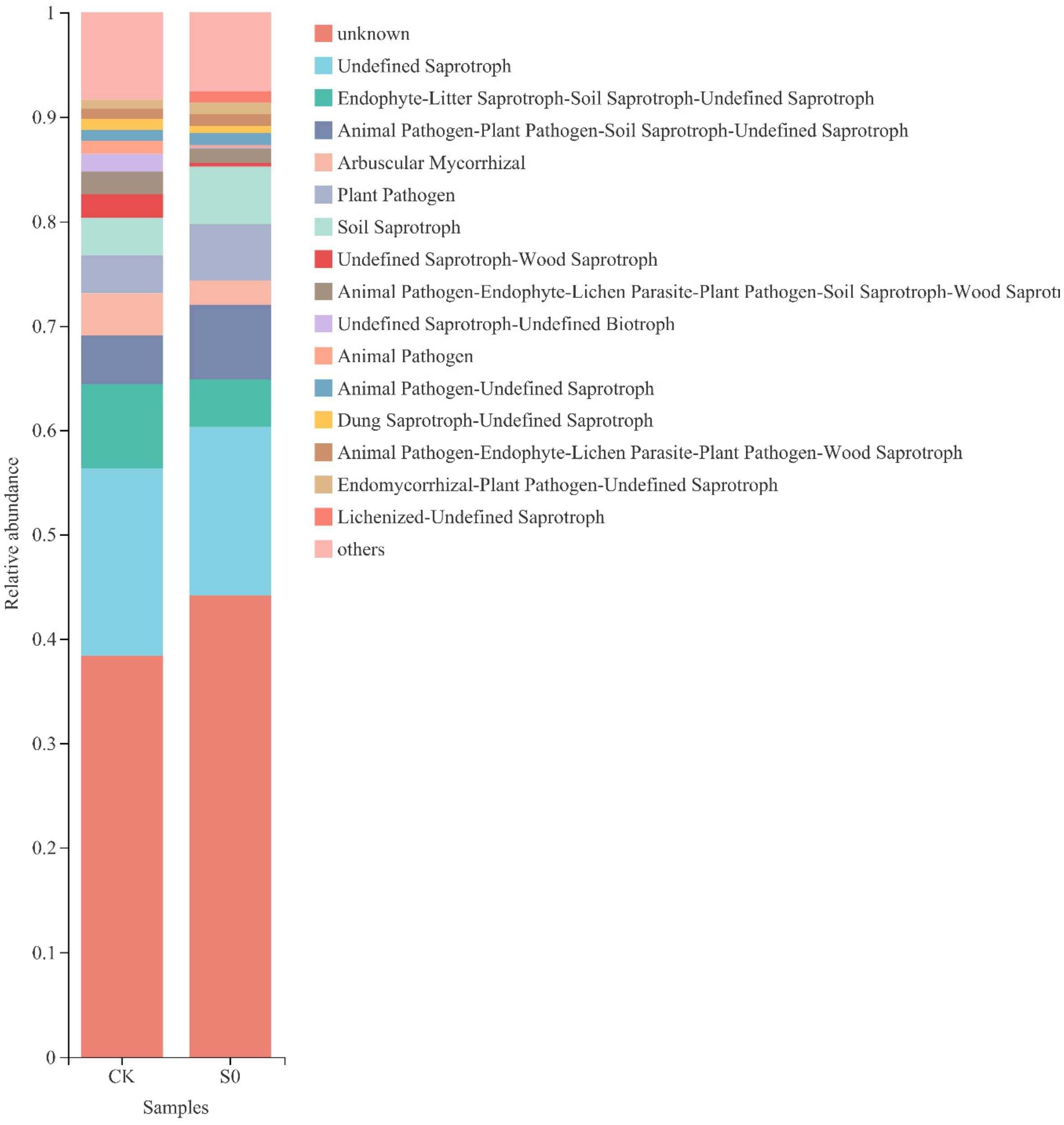

Figure S6. Variations in composition of fungal functional groups inferred by FUNGuild. FunGuild was used to annotate fungi with functional Guild; Saprophytes and pathogens were more responsive to snow change.
